# Audiological results and subjective benefit of an active transcutaneous bone-conduction device in patients with congenital aural atresia

**DOI:** 10.1007/s00405-021-06938-8

**Published:** 2021-06-26

**Authors:** Veronika Volgger, Inge Teresa Schießler, Joachim Müller, Florian Schrötzlmair, Marlene Pollotzek, John Martin Hempel

**Affiliations:** Department of Otorhinolaryngology, University Hospital, LMU Munich, Marchioninistr. 15, 81377 Munich, Germany

**Keywords:** Congenital aural atresia, Bonebridge, Bone conduction device, Hearing rehabilitation, SSQ

## Abstract

**Purpose:**

To review functional and subjective benefit after implantation of an active transcutaneous bone conduction device (BCD) in patients with congenital microtia with atresia or stenosis of the external auditory canal.

**Methods:**

Retrospective chart analysis and questionnaire on the subjective impression of hearing ( Speech, Spatial and Qualities of Hearing Scale (SSQ-B) of patients treated between 2012 and 2015.

**ResultsResults:**

18 patients (24 ears) with conductive or mixed hearing loss in unilateral (*n* = 10) or bilateral (*n* = 8) atresia were implanted with a BCD. No major complications occurred after implantation. Preoperative unaided air conduction pure tone average at 0.5, 1, 2 and 4 kHz (PTA 4 ) was 69.2 ± 11.7 dB, while postoperative aided PTA 4 was 33.4 ± 6.3 dB, resulting in a mean functional hearing gain of 35.9 +/- 15.6 dB. Preoperatively, the mean monosyllabic word recognition score was 22.9 % ± 22.3 %, which increased to 87.1 % +/- 15.1 % in the aided condition. The Oldenburger Sentence Test at S_0_N_0_ revealed a decrease in signal-to-noise-ratio from − 0.58 ± 4.40 dB in the unaided to − 5.67 ± 3.21 dB in the postoperative aided condition for all patients investigated. 15 of 18 patients had a subjective benefit showing a positive SSQ-B score (mean 1.7).

**Conclusion:**

The implantation of an active bone conduction device brings along subjective and functional benefit for patients with conductive or combined hearing loss.

## Introduction

Congenital aural atresia as a developmental disorder of the ear impairs hearing and can cause aesthetic deformities. Severe anomalies occur in around 1:10,000–20,000 newborns [1] and can appear isolated or as part of a syndrome (e.g., Goldenhar, Franceschetti). Besides cosmetic rehabilitation, if desired, functional rehabilitation should be provided even if the hearing impairment is unilateral. For toddlers with atresia, the choices for hearing rehabilitation are softband [like BAHA (Cochlear^™^, Melbourne, Australia), Ponto^™^ (Oticon Medical, Smørum, Denmark)] and conventional headband bone-conduction (BC) hearing aids. However, with increasing age, hearing rehabilitation is preferably achieved with active middle-ear implants or bone-conduction devices (BCD) [2]. Lately, there has been considerable interest in different BCDs available for hearing-impaired patients with unilateral or bilateral atresia. BCDs can be either passive or active devices, and can be either percutaneous or transcutaneous. Percutaneous devices have the disadvantage of being “open systems” and therefore bear a non-trivial risk of postoperative periabutment wound infections, occurring in approximately 5% of cases [3]. Even though the Osia^®^ 2 System (Cochlear^™^, Melbourne, Australia) was recently approved by the U.S. Food and Drug Administration (FDA) (2019), the only currently available active transcutaneous BCD on the European market and approved for both adults and children is the Bonebridge (MED-EL, Innsbruck, Austria).

Besides objective parameters in measuring the outcome of surgical procedures for hearing rehabilitation, it is very important to also assess the patients’ subjective hearing impression. Therefore, the aim of this study was to retrospectively analyze functional and subjective benefit after implantation of a Bonebridge in patients with atresia.

## Materials and methods

The study was approved by the local ethics committee and was conducted in compliance with the Declaration of Helsinki. For this retrospective clinical study, written informed consent was obtained from each participating individual (or his or her legal guardian). If the implantation of an active middle-ear device was not possible due to temporal bone anatomy or according to the family’s choice hearing rehabilitation was performed with a BCD (Bonebridge, MED-EL, Innsbruck, Austria). To assess severe malformations preoperatively, we used both the Jahrsdoerfer score [4] and the scoring system proposed by Frenzel et al. [5], which also considers the course of the facial nerve in relation to the round window. In patients with a Jahrsdoerfer grading score below 5, Bonebridge surgery was performed. 18 patients were included, of whom 10 had a conductive or combined hearing loss in unilateral atresia and 8 in bilateral atresia. 6 of the 8 patients with bilateral atresia were operated on both sides. Surgeries were performed between May 2012 and September 2015 at a single tertiary referral center by one experienced surgeon. Audiological inclusion criteria were a conductive or mixed hearing loss with BC thresholds up to 45 dB.

### Audiometry

Pre- and postoperative pure-tone audiometry with BC and air conduction (AC) thresholds was performed by professional staff for each side in the unaided and aided condition. Pure-tone average (PTA_4_) was calculated as the mean value of thresholds at 0.5, 1, 2, and 4 kHz. The functional hearing gain (FHG) was calculated comparing unaided AC PTA_4_ to aided PTA_4_. Speech understanding in quiet was tested using the German Freiburg test at 65 dB (monosyllables and numbers from a male speaker). Masking broad-band noise was presented at the contralateral ear with an AC headphone, when applicable. The speech reception threshold (50% correct) was determined with the Oldenburg Sentence test (OLSA) or the OLKISA test, if younger children were affected (*n* = 3; 5.3–6.9 years old). The OLSA noise (pseudo-continuous) served as the noise source. The test conditions involved presentation of speech and noise from the front (S_0_N_0_) and, for patients with unilateral atresia, presentation of speech from the front and noise to the contralateral side (S_0_N_-90_).

### Questionnaire

All 18 patients were retrospectively asked to answer a modified version of the “*Speech, Spatial and Qualities of Hearing Scale”* [6]*,* the SSQ-B questionnaire [7] (minors of 12 years with the help of their parents), which has been developed for the assessment of self-perceived improvement following an intervention. The SSQ-B questionnaire was mailed to the patients after surgery including a prepaid envelope for return. This validated questionnaire analyzes the subjective impression of hearing in environmental situations. It consists of 49 questions subdivided into questions that categorize on speech discrimination, spatial hearing, and hearing quality. In the SSQ-B, patients are asked whether the situation has changed as compared to preoperatively. Answers are selected from a rating scale (Likert scale ranging from -5 to 5). Positive scores indicate improvement, while negative scores indicate worsening. A score of 0 represents no change. Additionally, all patients were asked on how long they wore the audio processor (AP) every day.

Demographic data, audiometric results, surgery reports, and simultaneous or prior/later auricular reconstruction were obtained from medical charts.

## Results

Eighteen patients suffered from either unilateral (*n* = 10) or bilateral (*n* = 8) atresia. 6 of 8 patients with bilateral atresia were operated on both sides, resulting in a total of 24 operated ears. The relevant patients’ demographic and baseline data are summarized in Table 1. 10 patients underwent a simultaneous pinna reconstruction with a porous polyethylene framework (Medpor, Stryker, U.S.). Two patients had a canalplasty and 1 patient had a unilateral outer ear reconstruction elsewhere prior to BCD implantation. One﻿ patient had both a canalplasty and pinna reconstruction bilaterally prior to Bonebridge implantation in our clinic.

**Table 1:** Patients’ demographic and baseline data

	All patients	Unilat. disease Unilat. BB	Bilat. disease Bilat. BB	Bilat. disease Unilat. BB
*n* (Pt/ears)	18/24	10/10	6/12	2/2
Age	23 (5–54)	14 (5–54)	35 (11–53)	20 (17, 23)
M:F	8:10	6:4	1:5	1:1
Sim. pinna rec. (Pt/ears)	10/12	5/5	4/6	1/1
Prior canalplasty (Pt/ears)	3/4	1/1	2/3	0
Prior pinna rec. (Pt/ears)	2/3	1/1	1/2	0

*BB* Bonebridge, *Pt* patients, *M* male, *F* female, *Sim* simultaneous, *pinna rec* pinna reconstruction. Age is given as median with range in parentheses

The BCD implantation was performed without major surgical complications in all patients. The Bonebridge was either implanted in the sinudural angle of the mastoid (*n* = 21 ears) or using the middle fossa approach (*n* = 3 ears) [8]. In some cases, a slight impression of the dura (*n* = 2) or sigmoid sinus (*n* = 3) was necessary. 1 patient suffered from an air-filling chamber above the implant when blowing his nose postoperatively. This minor irregularity was resolved conservatively with a circular wrapped pressure bandage after a week. No further postoperative complications occurred.

### Audiological results

**Fig. 1:**
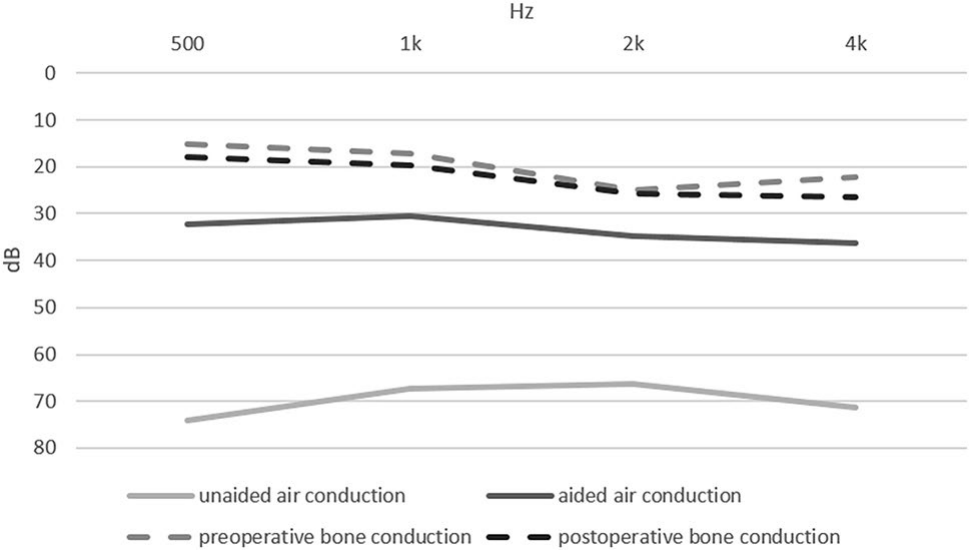
Preoperative unaided (light grey) and postoperative Bonebridge-aided (dark grey) pure-tone air conduction threshold (PTA_4_) as well as preoperative (grey, dotted) and postoperative (black, dotted) pure-tone bone-conduction threshold for all patients with atresia (*n* = 18). PTA_4_ = pure-tone average at 0.5, 1, 2 and 4 kHz

**Fig. 2:**
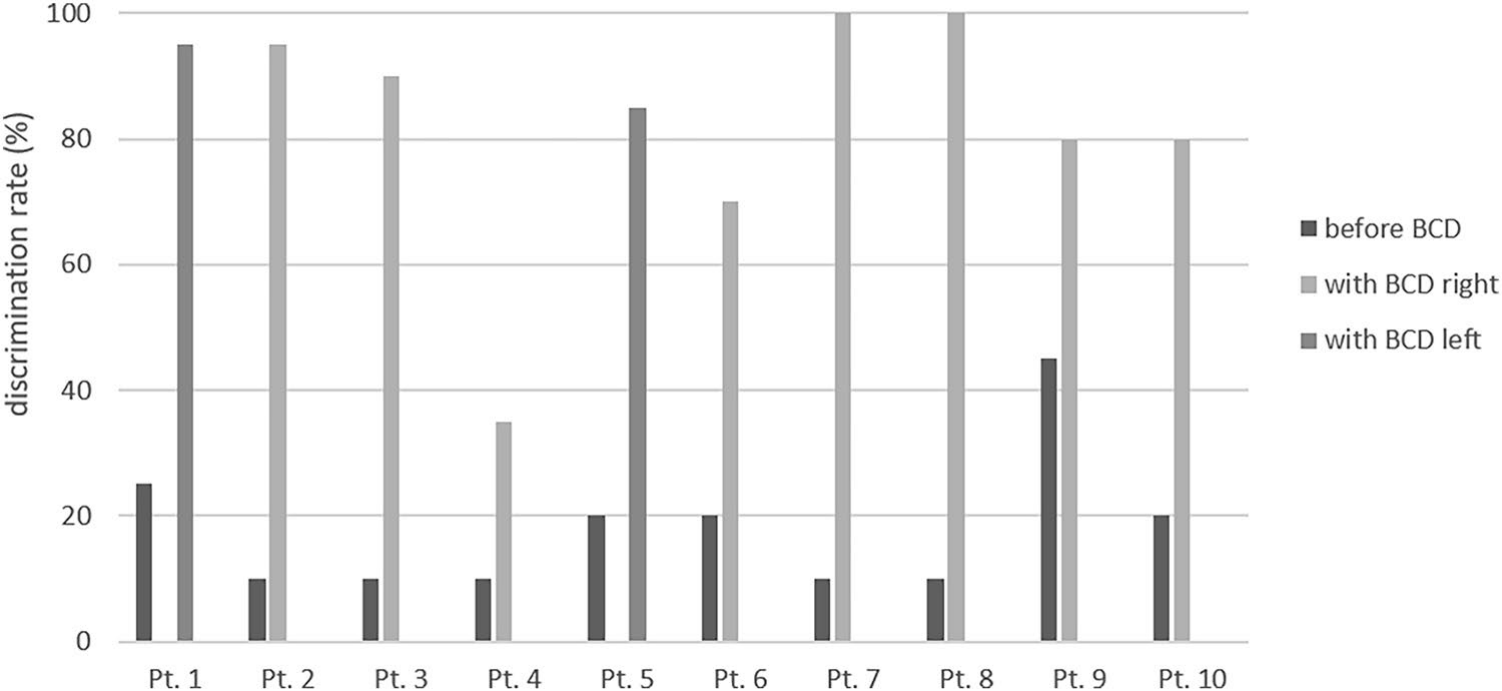
Discrimination rate tested with the German Freiburg test in a preoperative unaided and postoperative Bonebridge-aided condition for patients with unilateral atresia and unilateral implantation (*n* = 10)

**Fig. 3:**
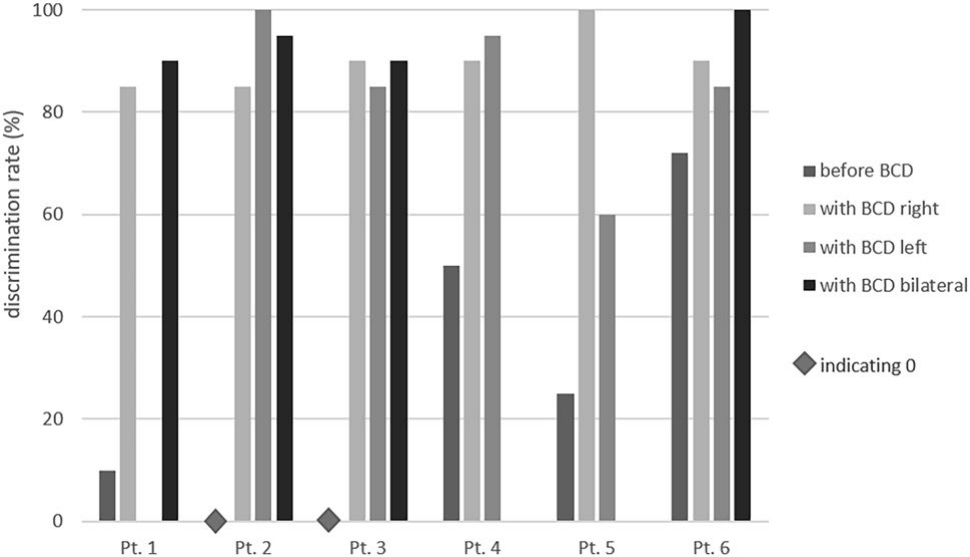
Discrimination rate tested with the German Freiburg test in a preoperative unaided and postoperative Bonebridge-aided condition for patients with bilateral atresia and bilateral implantation (*n* = 6)

14 ears (12 patients) showed a purely conductive hearing loss (with BC PTA_4_ < 20 dB), while the other 10 ears (9 patients) presented a mixed hearing loss. The detailed results of the pure-tone audiometry are depicted in Fig. 1. Table 2 shows both the results of the pure-tone audiometry as well as of the German Freiburg test. As on average, patients scored 87.1% ± 15.1% of monosyllables at 65 dB in the German Freiburg test, with 66.7% of patients (*n* = 12) scoring 90% or more. Fig. 2, 3 show the detailed results of the German Freiburg test for patients with unilateral atresia and unilateral BCD as well as for patients with bilateral atresia and bilateral BCD. For patients with bilateral atresia, results of the German Freiburg test were incomplete (see Fig. 3). Therefore, to calculate the mean postoperative aided speech discrimination rate, for the 2 patients, where the speech discrimination rate with bilateral BCD was missing, a mean out of the available results from the right and left side, respectively, was used.

**Table 2:** Results of pure-tone audiometry and German Freiburg test

	All patients	Unilat. disease Unilat. BB *n* = 10	Bilat. disease Bilat. BB *n* = 6	Bilat. disease Unilat. BB *n* = 2
Preop. BC [dB]	20.2 ± 6.1	18.1 ± 7.3	20.9 ± 3.6	25.4 ± 6.4
Preop. unaided AC [dB]	69.2 ± 11.7	71.7 ± 13.1	67.9 ± 11.6	66.3 ± 7.1
Preop. ABG [dB]	49.1 ± 10.6	53.6 ± 9.7	47.0 ± 9.2	40.9 ± 11.4
Postop. BC [dB]	23.1 ± 10.7	18.3 ± 9.8	25.3 ± 5.4	32.6 ± 15.1
Postop. aided AC [dB]	33.4 ± 6.3	34.4 ± 3.5	33.2 ± 8.4	29.9 ± 1.6
FHG [dB]	35.9 ± 13.2	36.8 ± 15.5	35.1 ± 13.1	36.4 ± 8.7
Preop. speech DR [%]	22.9 ± 22.3	18.0 ± 10.5	26.2 ± 26.8	37.5 ± 37.5
Postop. aided speech DR [%]	87.1 ± 15.1	83.0 ± 18.5	91.3 ± 6.1*	95.0 ± 5.0

*BC* bone conduction; *AC* air conduction; *ABG* air–bone gap; *FHG* functional hearing gain; *DR* discrimination rate. Preoperative measurements as indicated here were performed in the unaided condition

^*^bilaterally aided DR missing for two patients, for calculation, the mean of the speech DR of the left and right side was used

The OLSA was performed in a preoperative unaided und postoperative BCD-aided condition for all patients at 65 dB. Mean unaided signal-to-noise-ratio (SNR) for all atresia patients tested at S_0_N_0_ was  − 0.58 dB ± 4.40 dB, while mean aided SNR was  − 5.67 dB ± 3.21 dB (one dataset missing). For the subgroup of patients with unilateral atresia tested at S_0_N_0_, the mean unaided SNR was  − 2.23 dB ± 3.79 dB, while the mean aided SNR was  − 5.73 dB ± 2.87 dB. Three of the patients were tested with the OLKISA. Patients with bilateral atresia and bilateral BCD had a preoperative unaided mean SNR of 2.01 dB ± 4.37 dB and a postoperative mean SNR of  − 5.30 dB ± 2.10 dB, while patients with bilateral atresia and unilateral BCD had a preoperative mean SNR of  − 0.2 dB ± 3.7 dB and a postoperative mean SNR of  − 6.5 dB ± 6 dB when tested in the S_0_N_0_ condition. A further analysis of the test results for patients with unilateral atresia at S_0_N_-90_ was omitted, due to missing data for a relevant number of patients. However, for the sake of completeness, the results are listed in Table 3, along with the detailed results of the OLSA/OLKISA test at S_0_N_0_.

**Table 3:** OLSA and OLKISA results measured in a preoperative unaided and postoperative Bonebridge-aided condition

Pt. ID	Disease	BB	Test	S_0_N_0_		S_0_N_-90_	
				SRT_noise_ (dB SNR)		SRT_noise_ (dB SNR)	
				UA	BB	UA	BB
1	Unilat	Unilat	OLKISA	− 8.3	− 11.4	− 8.3	− 11.4
2	Unilat	Unilat	OLSA	− 3.8	− 4.4		
3	Unilat	Unilat	OLKISA	− 1.2	− 7		− 7
4	Unilat	Unilat	OLSA	− 2.1	− 6.7		
5	Unilat	Unilat	OLSA	6.5	− 0.9		
6	Unilat	Unilat	OLKISA			− 6.3	− 12.8
7	Unilat	Unilat	OLSA	− 3.8	− 4.2		
8	Unilat	Unilat	OLSA	− 0.6	− 8.5		− 13.7
9	Unilat	Unilat	OLSA	− 5	− 4.4		
10	Unilat	Unilat	OLSA	− 1.8	− 4.1	− 3.8	− 4
11	Bilat	Bilat	OLSA	− 1.1	− 5.7		
12	Bilat	Bilat	OLSA	8.0	− 2.4		
13	Bilat	Bilat	OLSA	0	− 8.5		
14	Bilat	Bilat	OLSA	− 0.8	− 7.3		
15	Bilat	Bilat	OLSA	7.6	− 4.1		
16	Bilat	Bilat	OLSA	− 3.1	− 3.8		
17	Bilat	Unilat	OLSA	3.5	− 0.5		
18	Bilat	Unilat	OLSA	− 3.9	− 12.5		

*Pt* patient, *dB* decibel, *BB* with Bonebridge, *UA* unaided, *S*_*0*_*N*_*0*_ speech and noise presented from the front, *S*_*0*_*N*_*-90*_ speech presented from the front, noise presented from the contralateral side, *SNR* signal-to-noise ratio, *SRT* speech reception threshold in noise

### Speech, spatial, and qualities of hearing scale

**Fig. 4:**
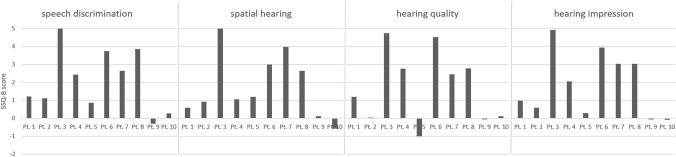
Bar diagram showing the Results of the Speech, Spatial, and Qualities of Hearing Scale (SSQ-B) for patients with unilateral atresia and unilateral implantation. Answers are selected from a Likert Scale ranging from  − 5 (maximal adverse effect) to 5 (maximal positive effect)

**Fig. 5:**
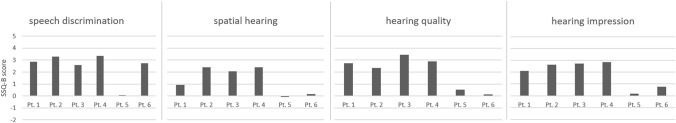
Bar diagram showing the Results of the Speech, Spatial, and Qualities of Hearing Scale (SSQ-B) for patients with bilateral atresia and bilateral implantation. Answers are selected from a Likert Scale ranging from  − 5 (maximal adverse effect) to 5 (maximal positive effect)

15 of 18 patients with atresia reported a benefit in overall hearing impression (mean 1.65; SD 1.6). For the subscores’ speech discrimination, spatial hearing, and hearing quality, the mean values were 1.94 ± 1.7, 1.46 ± 1.5, and 1.60 ± 1.8, respectively. All patients with bilateral implantation (*n* = 6) had a benefit in overall hearing impression with a mean score of 1.87 ± 1.1. 80% (8/10) of patients with unilateral atresia and unilateral BCD reported a benefit in overall hearing impression (mean score 1.87, SD 1.8), while patients with bilateral atresia and unilateral implantation (*n* = 2) reported an overall slight decrease in hearing impression (mean score -0.16). For patients with unilateral BCD in unilateral atresia, speech discrimination was a mean of 2.08 ± 1.7, spatial hearing 1.79 ± 1.7 and hearing quality 1.75 ± 2.0, while for patients with bilateral BCD in bilateral atresia, the scores were 2.48 ± 1.2, 1.32 ± 1.1, and 2.01 ± 1.4, respectively. The following subscores were reported from the 2 patients with bilateral atresia and unilateral BCD: speech discrimination 0.71/ − 1.41, spatial hearing 0.29/0.12, hearing quality  − 0.06/-0.65, and overall hearing impression 0.28/ − 0.60. Fig. 4 shows the detailed results for patients with unilateral atresia and unilateral BCD, while Fig. 5 shows the results for patients with bilateral BCD in bilateral atresia.

The AP was worn for an average of 12.7 h by the patients (median 12 h, range 6–24 h). Patients with unilateral atresia wore the AP for an average of 10.1 h (range 6–14.5 h), patients with bilateral BCD for an average of 15.3 h (range 12–24 h), and patients with unilateral BCD in bilateral disease for an average of 14.5 h (range 12–17 h).

## Discussion

BCDs have been used for decades in patients with conductive or mixed hearing loss with intolerance or inability to wear conventional hearing aids, and since the 2000s, their indication has been extended to single-sided deafness. The efficacy of percutaneous implants is well proved, but compared to transcutaneous devices, they more frequently cause skin problems, such as periabutment wound infections and overgrowing skin/soft tissue. In recent years, several reports on the use of transcutaneous devices, such as the Bonebridge, have been published and have shown promising results [9–12].

The aim of this study was to analyze subjective benefit and functional hearing results following Bonebridge implantation in patients with conductive and mixed hearing loss in atresia.

Hearing rehabilitation in patients with atresia can be performed in different ways—canalplasty with or without conventional hearing aid, active middle-ear implants, or different BCDs. Canalplasty, in our opinion, is nowadays not an option for the majority of patients, especially in severe deformities, due to the high rate of restenosis, infection, and limited audiological outcome [13, 14]. 3 patients in our study cohort had undergone canalplasty before, which did not lead to satisfactory results in any case. Due to recurrent infections, the patients could not wear conventional hearing aids. Active middle-ear devices as well as BCDs are well suited for most patients. Factors for deciding in favor of the one or the other are based on anatomical conditions and patients’ choices. If there is enough space in the middle ear to attach the floating mass transducer (FMT) to the ossicles or the round window (subjects with a preoperative Jahrsdoerfer score equal to or higher than 5) [4], an active middle-ear device, such as the Vibrant Soundbridge (MED-EL, Innsbruck, Austria), can be considered, with the advantage of a direct unilateral stimulation of the inner ear, thus ruling out the possibility of signal confusion. The disadvantage of active middle-ear devices is the more challenging surgery bearing, in principle, higher risks for patients. However, also BCDs have some minor limitations—a Bonebridge can only be implanted if the skull is thick enough. It is usually implanted in the mastoid but a positioning via a retrosigmoidal or a middle fossa approach is also possible [8]. Even though the surgery is considered easier, a number of uncommon but serious complications are known in the field, like the injury to the sigmoid sinus and the dura, as drilling takes place over the sinudural angle.

In the present study only patients who received a Bonebridge, either because of their anatomical preconditions or because of their individual choice, were analyzed. 10 of the 18 patients analyzed received a simultaneous pinna reconstruction with Medpor, which has only been rarely described in the literature [15, 16]. The Bonebridge was mostly placed in the sinudural angle of the mastoid (*n* = 21 ears). In 3 cases the Bonebridge had to be placed via the middle fossa approach above the temporalis line. The slight impression of the dura or sigmoid sinus has been described to be safe in previous studies [17] and did not cause any problems in our patients’ cohort as well and as expected. Simultaneous pinna reconstruction neither impeded surgical outcome of the Bonebridge implantation in any manner nor caused complications (data not shown).

The number of patients studied in the presented work is small, so when subdividing the patients into subgroups only small cohorts remain limiting the possibility to compare the outcome between groups. The retrospective study design bears some known limitations—first, some data might be missing, because recording was done without having in mind a follow-up retrospective data analysis. In the study at hand, pure-tone audiometry data were complete. This, however does not hold true for the German Freiburg test (Fig. 3) and the OLSA (Table 2). Second, the questionnaires were sent to the patients after treatment. Retrospectively answering questions about surgery bears the bias that patients might not remember everything exactly. Yet, trends can be read out from the data and results on larger patient groups, especially in atresia patients, are still missing in the literature.

### Audiological results.

Our audiometric data are comparable with other studies. The mean FHG was 35.9 dB for all patients in this study. Schmerber et al. reported a mean FHG of 26.1 dB for 16 patients [10], Ihler et al. of 33.6 dB for 6 patients [9], and Skarżyński et al. of 28.0 dB for 25 patients with conductive or mixed hearing loss after Bonebridge implantation [11]. An FHG of 35.4 dB was reported by Chan et al. after Bonebridge in 10 patients with atresia and simultaneous pinna reconstruction [16], of 34.8 dB by Wang et al. in 7 patients with simultaneous auricular reconstruction [15], and of 47.2 dB by Zernotti et al. in 14 patients with atresia [12]. These results are also comparable to other BCDs, where an FHG of 31.0 ± 8 dB was reported after implantation of an active transcutaneous BCD, being developed in cooperation between research groups at Chalmers University of Technology and Sahlgrenska University Hospital in Gothenburg, Sweden [18]. Similar results were reported after the implantation of the Sophono Alpha1 (38 dB) and the BAHA attract (41 dB), both being passive transcutaneous devices [19]. 10 of the implanted ears had a combined hearing loss. Sometimes BC thresholds came close to the limits of the indication spectrum of 45 dB. In those cases, the expectations regarding the FHG are limited. This was thoroughly discussed with the patients concerned before surgery. Yet, the vast majority of patients profited from surgery, even in audiological borderline indications. Speech discrimination in quiet by Freiburg monosyllable test showed a clear improvement following BCD implantation (mean increase 64.2%) reaching 87.1%, which compares nicely to the current literature describing improvements of 21 to 63.3% and attaining up to 95% speech understanding in quiet [9, 10]. Also in this case, a higher score was not to be expected with a relevant number of patients with combined hearing loss. The 2 missing speech discrimination results in patients with bilateral implantation, to our opinion, do not distort the reported results, as a mean value out of the separately reported results for the right and left side was used instead. This approach was considered to best estimate and rather undervalue than overvalue the real benefit in speech discrimination of a bilateral BCD. In the OLSA test (S_0_N_0_), the mean SNR ratio decreased from  − 0.6 ± 4.4 dB to  − 5.7 ± 3.2 dB when looking at the whole study population, thus marking an improvement in speech understanding in noise for all except one patient and coming close to normal test values ( − 7.1 ± 1.1 dB). The variety of possible protocols makes a comparison of our data with the literature difficult. Furthermore, within this study, data were missing for 1 patient for S_0_N_0_, while for S_0_N_-90_, only very few data were available, thus impeding the conclusion drawn from the available data. A prospectively designed study could overcome this problem and deliver comparable and complete audiometric data. Rahne et al. reported an improvement from  − 2.3 dB SNR (preoperative best aided condition) to  − 3.3 dB SNR for S_0_N_0_ after Bonebridge for 11 patients with conductive or mixed hearing loss caused by middle-ear disorders or atresia of the ear canal. When the protocol was changed to S_90_N_-90_, the SNR improved from 1.3 dB to  − 6.1 dB [20]. Reinfeldt published an SNR with a transcutaneous BCD of  − 5.5 ± 2.3 dB for S_0_N_0_ in 6 patients with conductive or mixed hearing loss [18]. Thus, our results compare nicely to the current literature and reflect the patients reported subjective benefits.

### Subjective patient’s benefit

There was a 100% rate of return for SSQ-B questionnaires, which is higher than expected when compared to the current literature (about 80%) [21, 22]. 15 of the 18 patients achieved scores > 0, indicating a subjective improvement when compared to the pre-surgical hearing condition. The mean score for all patients was 1.65. For the subgroups bilateral atresia and bilateral BCD, as well as unilateral atresia and unilateral BCD, the mean hearing impression was 1.87, while it was clearly lower in patients with bilateral atresia and unilateral Bonebridge. This lower score in the group of patients with bilateral disease and unilateral BCD was caused by a patient with CHARGE-syndrome, with unilateral atresia on one and cup ear deformity (Weerda II) in severe outer ear canal stenosis on the other side. In the ear, hearing aids were not working due to the stenosis, while behind the ear hearing aids, even though satisfactory from an auditory viewpoint, did not work, as they continued to fall off. The patient rejected auricular reconstruction. The patient was not satisfied with the Bonebridge, probably because he was used to the sound as well as the stronger amplification of a conventional hearing aid. This case should teach us that the switch from a conventional hearing aid to a BCD is difficult, and that such patients should be given implants only with extreme caution. With only 2 patients remaining within this group, it is impossible to make a general statement on performance of patients with bilateral atresia and unilateral BCD—the second patient was performing well.

However, it is also worth looking at more patients individually. In the group of patients with unilateral atresia, 2 patients showed negative scores for overall hearing impression in the SSQ-B questionnaire. Both patients (16 years, 54 years old) had no hearing experience prior to surgery with hearing aids, except for a preoperative test phase of 3 months with conventional BC hearing aids. This may explain the limited outcome scores with long-term deprivation. Nevertheless, they both wear the device. However, it should be mentioned at this point that the information given by the patients on how long they wear the AP every day is subjective and was not counterchecked.

When carefully looking at subgroups in this small cohort, patients with bilateral BCD performed slightly better than patients with unilateral BCD in speech discrimination and hearing quality, while patients in unilateral atresia benefitted more regarding spatial hearing. This might be by chance in such small groups. Yet, if looking for an explanation for the slightly worse performance regarding speech discrimination and hearing quality in patients with unilateral atresia compared to patients with bilateral disease, a certain co-stimulation of the contralateral healthy ear, which seems not to be that important in patients with bilateral disease, might be the reason. Overall, the gained results indicate an improvement for most patients when compared to the pre-surgical hearing, which is also supported by the fact that all patients continue to wear and use the BCD. However, the results need to be looked at with caution as 1) the questionnaire was answered by the patients only once, thus representing the subjective hearing impression at that special moment, and being possibly influenced by the patients’ mood and 2) because a comparison group, e.g., patients wearing conventional BC hearing aids, is missing. Nevertheless, the results of the SSQ-B questionnaire seem reasonable. After implantation of the Sophono Alpha 1 in unilateral mixed hearing loss, bilateral mixed hearing loss and bilateral conductive hearing loss, values of approximately 1.25, 0.40, and 1.65 were reported for overall hearing impression [23].

## Conclusion

Patients with conductive or combined hearing loss in atresia had both a subjective and functional benefit from Bonebridge implantation with results comparable to other BCDs. The implantation, even when performed simultaneously to pinna reconstruction with a porous polyethylene framework, did not cause any severe complications requiring revision surgery. Both patients with unilateral and bilateral disease seem to have a benefit from BCD implantation.
